# Immunomodulatory Effects of RAAS Inhibitors: Beyond Hypertension and Heart Failure

**DOI:** 10.3390/biomedicines13071779

**Published:** 2025-07-21

**Authors:** Raluca Ecaterina Haliga, Elena Cojocaru, Oana Sîrbu, Ilinca Hrițcu, Raluca Elena Alexa, Ioana Bianca Haliga, Victorița Șorodoc, Adorata Elena Coman

**Affiliations:** 1 Internal Medicine and Toxicology Department, Faculty of Medicine, “Grigore T. Popa” University of Medicine and Pharmacy, 700115 Iasi, Romania; raluca.haliga@umfiasi.ro (R.E.H.); raluca-elena.alexa@umfiasi.ro (R.E.A.); victorita.sorodoc@umfiasi.ro (V.Ș.); 2 2nd Internal Medicine Department, St. Spiridon Clinical Emergency Hospital, 700115 Iasi, Romaniaelena.coman@umfiasi.ro (A.E.C.); 3 Morpho-Functional Sciences II Department, Faculty of Medicine, “Grigore T. Popa” University of Medicine and Pharmacy, 700115 Iasi, Romania; 4 Faculty of Medicine, “Grigore T. Popa” University of Medicine and Pharmacy, 700115 Iasi, Romania; mg-rom-30099@students.umfiasi.ro; 5 Preventive Medicine and Interdisciplinarity Department, Faculty of Medicine, “Grigore T. Popa” University of Medicine and Pharmacy, 700115 Iasi, Romania

**Keywords:** RAAS inhibition, immunomodulation, ACE inhibitors, neuroprotection, tumor microenvironment

## Abstract

The renin–angiotensin–aldosterone system (RAAS) plays a central role in cardiovascular and renal homeostasis and is increasingly recognized for its broad immunomodulatory effects. Pharmacological RAAS inhibition, primarily via angiotensin-converting enzyme inhibitors (ACEIs) and angiotensin receptor blockers (ARBs), has demonstrated therapeutic value beyond its use in hypertension and heart failure, extending to autoimmune, infectious, oncologic, and neurodegenerative conditions. ACEIs and ARBs modulate both innate and adaptive immune responses through Ang II-dependent and -independent mechanisms, influencing macrophage polarization, T-cell differentiation, cytokine expression, and antigen presentation. Notably, ACEIs exhibit Ang II-independent effects by enhancing antigen processing and regulating amyloid-β metabolism, offering potential neuroprotective benefits in Alzheimer’s disease. ARBs, particularly telmisartan and candesartan, provide additional anti-inflammatory effects via PPARγ activation. In cancer, RAAS inhibition affects tumor growth, angiogenesis, and immune surveillance, with ACEIs and ARBs showing distinct yet complementary impacts on tumor microenvironment modulation and chemotherapy cardioprotection. Moreover, ACEIs have shown promise in autoimmune myocarditis, colitis, and diabetic nephropathy by attenuating inflammatory cytokines. While clinical evidence supports the use of centrally acting ACEIs to treat early cognitive decline, further investigation is warranted to determine the long-term outcomes across disease contexts. These findings highlight the evolving role of RAAS inhibitors as immunomodulatory agents with promising implications across multiple systemic pathologies.

## 1. Introduction

**Figure 1:**
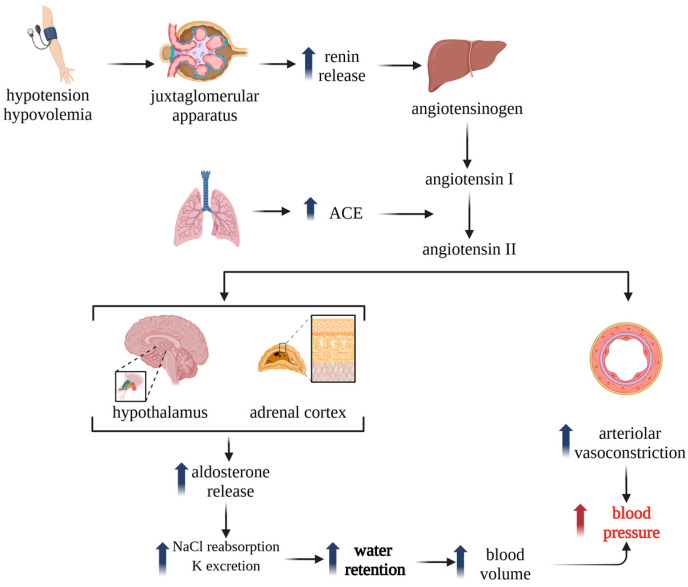
RAAS-mediated regulation of blood pressure and fluid volume (adapted from [3]). (Created with www.BioRender.com; accessed on 26 June 2025).

The renin–angiotensin–aldosterone system (RAAS) is a fundamental neurohormonal cascade that maintains cardiovascular and renal homeostasis by regulating the arterial pressure, fluid volume, and electrolyte balance. The activation of the RAAS, typically triggered by reduced renal perfusion, sympathetic nervous system stimulation, or sodium depletion, initiates renin release from the juxtaglomerular cells of the kidney. Renin cleaves hepatic angiotensinogen to generate angiotensin I (Ang I), which is subsequently converted by angiotensin-converting enzyme (ACE), predominantly located in the pulmonary endothelium, into angiotensin II (Ang II) (Figure 1). Ang II exerts potent vasoconstrictive, aldosterone-stimulating, and sodium-retentive effects, primarily through the Ang II type 1 receptor (AT1R). Persistent RAAS activation promotes oxidative stress, vascular remodeling, endothelial dysfunction, and fibrosis, key features of hypertensive and cardiorenal pathology [1,2].

Given the pivotal role of Ang II in the pathogenesis of hypertension and heart failure (HF), the pharmacological inhibition of the RAAS has become a cornerstone of the therapies for these conditions. Angiotensin-converting enzyme inhibitors (ACEIs) lower the Ang II concentrations, leading to vasodilation, a reduced afterload, and enhanced natriuresis, thereby mitigating pressure overloading and the long-term cardiovascular risk. Additionally, ACEIs increase the levels of angiotensin 1–7 (Ang-(1–7)), which binds the Mas receptor and exerts vasodilatory and anti-inflammatory effects, contributing to endothelial protection [4,5,6].

RAAS activation in HF is a compensatory mechanism that becomes maladaptive over time. A reduced cardiac output leads to renal hypoperfusion and sustained RAAS stimulation, promoting fluid retention, myocardial hypertrophy, and fibrosis. ACEIs have been shown to attenuate these deleterious processes by mitigating the preload and afterload, improving ventricular remodeling and enhancing the survival outcomes. Landmark clinical trials have consistently demonstrated reductions in the mortality and hospitalization rates in patients with HF with a reduced ejection fraction (HFrEF), supporting the inclusion of ACEIs as first-line agents in HF management guidelines [7,8,9].

Beyond their cardiovascular benefits, RAAS inhibitors, particularly ACEIs, have demonstrated important immunomodulatory effects. By reducing the Ang II levels, ACEIs suppress pro-inflammatory signaling pathways, such as those related to nuclear factor kappa B (NF-κB) activation and cytokine release, thus influencing both innate and adaptive immune responses [1,2]. Additionally, the shift toward increased angiotensin (1–7) and reduced aldosterone activity promotes an anti-inflammatory environment [6]. These immunological effects open new therapeutic perspectives for using RAAS inhibitors in autoimmune, inflammatory, and infectious diseases, topics that will be explored in the following sections [1,2].

Centrally acting ACE inhibitors (C-ACEIs), such as perindopril, fosinopril, trandolapril, and ramipril, can cross the blood–brain barrier and may influence the central RAAS activity and neuroinflammation [10,11]. In contrast, non-centrally acting ACEIs (NC-ACEIs) like enalapril, benazepril, and lisinopril act primarily in peripheral tissues [12]. ARBs (e.g., telmisartan, losartan) selectively block AT1 receptors, with some agents also activating PPARγ, thereby exerting broader anti-inflammatory effects [13]. Direct renin inhibitors and mineralocorticoid receptor antagonists (MRAs) have distinct mechanisms within the RAAS cascade [14] (Table 1).

## 2. Immunomodulatory Effects of ACEIs and ARBs

### 2.1. ACE and Immune Modulation: From Blood Pressure to Immunity

Initially developed to treat hypertension, ACEIs have since been recognized for their broader biological effects, notably in immune regulation. The link between ACE and immunity was first suggested in 1975, when elevated serum ACE levels were found in most patients with active sarcoidosis [18]. In granulomatous diseases, ACE expression by epithelioid macrophages and multinucleated giant cells implies a functional role in the host’s defense against persistent pathogens, including *Mycobacterium tuberculosis* and certain fungi [19,20].

**Figure 2:**
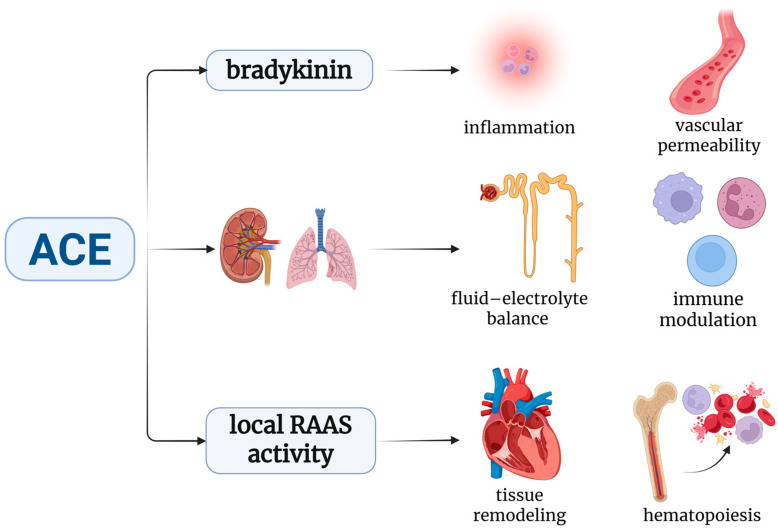
Pleiotropic effects of ACE beyond RAAS regulation [21,22]. (Created with www.BioRender.com; accessed on 26 June 2025).

Although ACE is classically known for its role in the RAAS, converting Ang I into the active peptide Ang II, its physiological functions extend far beyond vascular homeostasis. ACE is, in fact, a ubiquitous enzyme with a broad substrate specificity, implicated in a wide array of physiological processes beyond blood pressure regulation. ACE also degrades bradykinin, thereby modulating the vascular permeability and inflammation. It is expressed in multiple tissues, including the kidneys, lungs, and immune cells, contributing to maintaining the fluid–electrolyte balance, renal autoregulation, and immune modulation. Additionally, ACE influences the local RAAS activity related to tissue remodeling, hematopoiesis, and cognitive function (Figure 2) [21,22]. The biological effects of ACE can be broadly categorized into Ang II-dependent and Ang II-independent pathways (Table 2) [23,24].

Its Ang II-dependent effects include the AT1 receptor-mediated promotion of inflammation, macrophage activation, and pro-inflammatory cytokine production (e.g., the production of *tumor necrosis factor*-alpha (TNF-α), interleukins (IL-6), and interferon (IFN-γ)). Ang II also upregulates endothelial adhesion molecules (E-selectins, vascular cell adhesion molecule-1 (VCAM-1), intercellular adhesion molecule-1 (ICAM-1)) and influences dendritic cells’ migration and maturation, while impairing their phagocytic function. Th1 cells produce interferon-γ (IFN-γ) and support cell-mediated immunity, while Th17 cells secrete interleukin-17 (IL-17), contributing to tissue inflammation and autoimmunity. The polarization of naïve CD4^+^ T-cells into Th1 or Th17 subsets is driven by distinct cytokine environments and is critical for mounting specific immune responses. This process, when dysregulated, is enhanced by Ang II–AT1R signaling and sustains chronic inflammatory conditions, such as colitis and myocarditis [23,25,26,27].

ACE’s Ang II-independent effects involve its broad peptidase activity, including the hydrolysis of amyloid-β1–42, critical in Alzheimer’s disease [28]. Furthermore, ACE modulates antigen processing and presentation via major histocompatibility complex (MHC) class I pathways in antigen-presenting cells (APCs), including macrophages and dendritic cells [24,29]. An ACE deficiency in murine models has been associated with a range of impairments, including hypotension, impaired renal development and function, reduced male fertility, and abnormal myelopoiesis. These phenotypes underscore the enzyme’s essential role in vascular homeostasis, nephrogenesis, reproductive physiology, and hematopoietic regulation, functions that are not rescued by other carboxypeptidases [21,23,30].

Immune cells, including T lymphocytes, express the local components of a “lymphocytic RAAS”, where Ang II acts as both a cytokine and chemoattractant. A critical immunological balance is maintained by the contrasting effects of AT1Rs (pro-inflammatory) and AT2Rs (anti-inflammatory effects via nitric oxide pathways) [31,32,33,34].

Similarly, angiotensin receptor blockers (ARBs) exert both Ang II-dependent and -independent immunomodulatory effects. ARBs inhibit AT1R-mediated pro-inflammatory signaling (NF-κB and AP-1 pathways) and, through PPARγ activation, modulate lipid metabolism and insulin sensitivity and suppress inflammation. Agents like telmisartan, candesartan, and, to a lesser extent, losartan act as partial PPARγ agonists, providing additional anti-inflammatory benefits [31]. Not all ARBs exert significant PPARγ activity. Telmisartan is a strong partial agonist of PPARγ, and candesartan exhibits moderate activity, while losartan displays minimal or inconsistent PPARγ activation. This pharmacodynamic variability may contribute to their differential anti-inflammatory effects [16,31].

**Table 2:** Biological effects of ACE.

Biological Effects of ACE	Mechanisms	Effects	Experimental Model	RAAS Inhibitor Used	References
Ang II-dependent	AT1R activation → NF-κB → increases IL-6, TNF-α	Promotes Th17 polarization; inflammatory cytokine production	DSS-induced colitis in mice	Telmisartan	[35]
Ang II-dependent	AT2R activation → NO release → reduces inflammation	Anti-inflammatory, vasodilatory, and antifibrotic effects	Endothelial cell culture under shear stress	Candesartan	[36]
Ang II-independent	ACE hydrolyzes Aβ1–42 → modulation of amyloid burden	Neuroprotective in AD models	APP/PS1 transgenic mice	Ramipril	[37]
Ang II-independent	MHC class I peptide trimming by ACE → augment antigen presentation	Enhanced CD8^+^ T-cell activation	ACE 10/10 mice	Captopril	[24,38]

DSS—dextran sulfate sodium; AD—Alzheimer’s disease; APP/PS1—transgenic mouse model of AD.

### 2.2. Effects of RAAS Inhibitors on Innate Immunity

#### 2.2.1. Modulation of Macrophage and Dendritic Cell Function

The immunological role of ACE was convincingly demonstrated using ACE 10/10 mice, transgenic models with their ACE expression restricted to myeloid cells and knocked out in all other tissues. This allowed for the selective investigation of ACE’s immune functions, revealing its role in enhancing antigen presentation and pro-inflammatory responses, independent of its vascular effects. The designation “10/10” has no biological significance and was assigned by the original developers as a transgenic line label [19,24,34]. Originally designed for the study of atherosclerosis, this model revealed that macrophages with an increased ACE expression predominantly adopted a pro-inflammatory M1 phenotype. These macrophages exhibited the enhanced production of IL-12 and nitric oxide (NO) and reduced IL-10 secretion, contributing to improved pathogen resistance and anti-tumor responses [23,34].

ACE overexpression enhances both innate and adaptive immune responses, notably increasing CD8^+^ T-cell activation and IgG1 antibody production. These effects, demonstrated in ACE 10/10 transgenic mice with myeloid-specific ACE expression, are reversed by ACE inhibitors but unaffected by ARBs, confirming an Ang II–independent immunomodulatory function of ACE [29,37].

Experimental tumor models have shown that ACE 10/10 mice develop significantly smaller tumors compared to wild-type controls. Furthermore, enhanced bacterial clearance following infection with *Listeria monocytogenes* and *Methicillin-Resistant Staphylococcus aureus* (MRSA) was observed, underscoring ACE’s role in boosting innate immunity [19,24,39,40].

ARBs, such as losartan, telmisartan, and candesartan, have demonstrated context-dependent immunomodulatory properties. While losartan may paradoxically promote macrophage activation via LOX-1 upregulation [41], candesartan has been shown to induce an anti-inflammatory M2-like microglial phenotype and enhance β-amyloid clearance in Alzheimer’s disease models [42] (Table 3).

#### 2.2.2. Impact on Neutrophil Activation and Oxidative Stress

Neutrophils rely on NADPH oxidase (NADPHox)-mediated reactive oxygen species (ROS) production for effective microbial killing. ACE enhances ROS generation and bacterial clearance, while an ACE deficiency impairs neutrophil function, resulting in an increased infection burden [39]. Importantly, ACE inhibition abrogates these protective effects, raising concerns regarding its impact on the perioperative infection risk [43]. ACEIs may therefore dampen neutrophil activation by attenuating bactericidal ROS generation, potentially compromising innate immune responses to bacterial infections. Experiments on animal models support this, demonstrating impaired neutrophil chemotaxis and phagocytosis following ACEI therapy [31,39].

ARBs, by activating PPARγ and suppressing oxidative stress, may also blunt the neutrophil-driven microbial clearance. Nonetheless, some ARBs such as candesartan have shown intrinsic antimicrobial activity against *Staphylococcus aureus* and *Candida albicans*, indicating a more complex interplay between immunomodulation and host defense [44,45,46] (Table 3).

**Table 3:** Differential effects of ACEi and ARBs on innate immunity.

Immune Function	Effects of ACEi	Effects of ARBs
Macrophage polarization	Inhibits M1 phenotype (pro-inflammatory); reduces IL-12 and NO; increases IL-10; suppresses pro-inflammatory activity (captopril) [34,38]	Context-dependent: may promote M1 (pro-inflammatory) phenotype via LOX-1 (losartan) or induce M2 (anti-inflammatory) phenotype (candesartan) [41,42]
Dendritic cell and CD8^+^ T-cell activation	Reduces activation (reverses ACE overexpression effects) [37]	No significant effect on activation seen in ACE 10/10 model
Tumor growth (ACEIs) and bacterial clearance	Suppresses enhanced resistance seen with ACE overexpression [39,40]	Decreased risk of cancer overall and several site-specific cancers [46]
Neutrophil function and ROS production	Reduces NADPHox-mediated ROS; impairs bacterial killing [39,43]	Reduce ROS via PPARγ; may compromise neutrophil function; intrinsic antimicrobial activity (e.g., candesartan) [44,45]

These findings should not discourage the use of RAAS inhibitors where clinically indicated. Rather, they emphasize the need for individualized risk–benefit assessments, particularly in patients with an active infection or immunosuppression or in perioperative settings where the neutrophil function is critical for recovery and defense.

### 2.3. Effects of RAAS Inhibitors on Adaptive Immunity

#### 2.3.1. Influence on T-Cell Differentiation

Ang II promotes the differentiation of pro-inflammatory Th1 and Th17 subsets and the production of IFN-γ and TNF-α. ACE inhibition counteracts these effects, as shown in experimental lung and renal models, where captopril and enalapril reduced the inflammatory cytokine levels and restored the T-cell balance [47,48].

The Th1/Th2/Th17/Treg balance is crucial in immune regulation. RAAS activation skews T-cell differentiation toward the Th1 and Th17 subsets via IL-12 and IL-6 signaling, while ACEIs suppress this polarization [49,50]. Th2 responses may also be modulated by ARBs, particularly in allergic models [51]. Emerging data suggest that RAAS inhibitors can indirectly promote Treg expansion, enhancing immune tolerance, although the results vary by the agent and disease model [1,48,51,52].

ARBs further modulate the T-cell activity by suppressing calcium-dependent signaling pathways (via Kv1.3 and KCa3.1 channel inhibition) [53]. Kv1.3 channels maintain the membrane potential in activated T-cells by facilitating a K^+^ efflux, enabling a sustained Ca^2+^ influx through Ca^2+^ release-activated Ca^2+^ (CRAC) channels. This calcium entry is essential for downstream signaling and cytokine production; thus, Kv1.3 indirectly supports Ca^2+^-dependent T-cell activation and Th1/Th17 polarization [31,54]. In myocardial infarction models, valsartan restored the Kir2.1 expression, protecting against arrhythmias through immune modulation [55]. Additionally, ARBs have shown beneficial effects in allergic lung diseases by reducing the Th2 cytokines and inhibiting mast cell degranulation [51].

The influence of ARBs on regulatory T-cells (Tregs) remains variable; fimasartan appears to increase Tregs [56], while losartan decreased the Treg levels in a *Plasmodium berghei* infection model, possibly reflecting a pathogen-specific immune response [57].

#### 2.3.2. Modulation of B-Cell Function and Antibody Production

Although ACE is not expressed in B lymphocytes, ACEIs can indirectly modulate humoral immunity by affecting T-cell–B-cell interactions. In experimental models, enalapril reduced the IgG production post-myocardial infarction and attenuated the B-cell infiltration in nephropathy models [48,52,58].

### 2.4. Cytokine Regulation and Inflammatory Pathways

ACEIs have demonstrated significant immunomodulatory effects, particularly through the attenuation of pro-inflammatory cytokine production and an increase in anti-inflammatory mediators. Notably, their immunomodulatory effects may vary with the dosage. For instance, while low-dose captopril reduces TNF-α and IL-1β [59], high doses in murine models have paradoxically induced depressive-like behaviors and reduced the Treg populations [60], suggesting potential pro-inflammatory shifts at supratherapeutic concentrations.

In patients with chronic heart failure, especially those with cachexia, elevated TNF-α levels in peripheral blood mononuclear cells are common. Captopril has been shown to suppress TNF-α production by up to 74% and reduce IL-1 synthesis by nearly 60%, contributing to its therapeutic benefit in this cohort [2,22,61]. Beyond its systemic effects, captopril also enhances the expression of IL-10 and TGF-β in cardiac tissue, particularly following myocardial infarction or in hypertensive patients [38,62]. It increases the IL-1 receptor antagonist levels, thereby promoting an anti-inflammatory milieu and improving immune homeostasis [2]. Comparable cytokine-suppressive properties have been reported for other ACEIs, including lisinopril, enalapril, and perindopril [63,64]. Moreover, captopril reduces the inflammatory markers in non-cardiac conditions, such as hepatic fibrosis and acute pancreatitis [65]. However, the immune responses to ACEIs may vary by the disease context. For instance, in psychiatric disorders, captopril has been associated with increased IL-1β and IL-6 levels, reduced regulatory T-cell (Treg) populations, and behavioral changes indicative of microglial activation [2,60,66].

In murine colitis, enalapril significantly reduced the intestinal inflammation by lowering the TNF-α, IFN-γ, IL-6, IL-8, and IL-1β expression, as well as by reducing the immune cell infiltration into colonic tissue [67]. Similar protective effects were observed in inflammatory lung injury models, where enalapril reduced the IL-1β and IL-6 levels in the respiratory tract, although systemic inflammatory markers and vascular remodeling remained unchanged [68]. In the context of diabetic nephropathy, enalapril inhibited the TNF-α mRNA expression in the renal cortex and decreased both the renal and urinary TNF-α concentrations, correlating with reduced albuminuria and suggesting a protective effect against inflammation-induced renal damage [2,69]. Furthermore, RAAS inhibition has demonstrated beneficial immunomodulatory effects in preclinical models of autoimmune myocarditis and multiple sclerosis, indicating potential therapeutic roles in conditions beyond cardiovascular and renal pathology [70,71].

**Figure 3:**
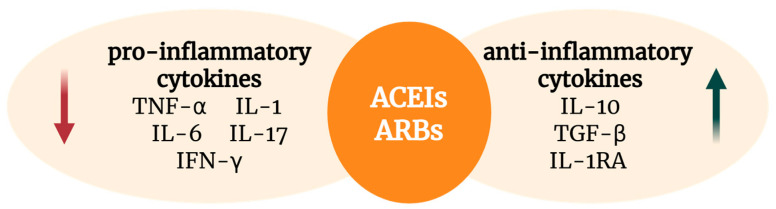
Effects of RAAS inhibitors on cytokine profiles. (Created with www.BioRender.com; accessed on 26 June 2025).

ARBs have also been shown to downregulate several pro-inflammatory cytokines, including TNF-α, IL-1, IL-6, IFN-γ, and IL-17. While the IL-4 expression is typically unaffected, some pathological contexts may lead to increased levels of IL-4 and IL-5 [2]. Additional studies have reported reductions in IL-5, IL-8, and IL-13 following ARB administration [51,53,66]. ARBs may also enhance the profiles of anti-inflammatory cytokines, particularly IL-10 and TGF-β [72] (Figure 3), although the findings regarding their TGF-β modulation remain inconsistent [31].

### 2.5. Key Signaling Pathways in RAAS–Immune System Crosstalk

The immunomodulatory activity of RAAS components is mediated through key intracellular pathways. Ang II–AT1R activation promotes NF-κB translocation, triggering the transcription of pro-inflammatory cytokines like IL-6, IL-1β, and TNF-α. It also activates MAPK cascades (ERK1/2, JNK), contributing to cellular stress responses and cytokine expression. Additionally, Ang II can activate the JAK-STAT pathway, influencing Th1 polarization. An RAAS blockade may also influence the NLRP3 inflammasome, with ACEIs like enalapril shown to suppress inflammasome activation in nephropathy models [48].

### 2.6. Comparative Immunomodulatory Profile: ACEIs vs. ARBs

**Figure 4:**
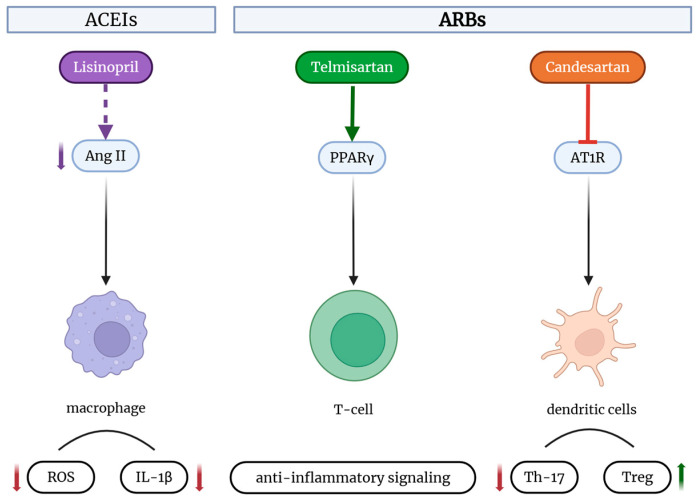
Comparative immunomodulatory effects of ACEIs and ARBs on immune cells. (Created with www.BioRender.com; accessed on 26 June 2025). Lisinopril (ACEI) reduces Ang II levels, leading to decreased ROS and IL-1β production in macrophages. Telmisartan (ARB) activates PPARγ in T-cells, promoting anti-inflammatory signaling. Candesartan (ARB) blocks AT1R signaling in dendritic cells, decreasing Th17 differentiation and increasing Treg responses [2,77].

While both ACEIs and ARBs modulate immune responses, they do so via distinct mechanisms. ACEIs reduce Ang II synthesis and bradykinin degradation, promoting vasodilation and an increase in Tregs, but may impair neutrophil function [73,74]. ARBs block AT1Rs directly and modulate PPARγ pathways, enhancing M2 macrophage polarization and Th17 suppression [31] (Figure 4). The clinical implications differ: ACEIs are preferred for treating autoimmune myocarditis [75], whereas ARBs may be superior in treating neuroinflammation and allergic conditions [51,76]. Their relative efficacy may also depend on their tissue penetration and pharmacokinetics [2,31].

### 2.7. Adverse Effects and Safety Considerations

Despite the promising immunomodulatory effects of RAAS inhibitors, their clinical use is associated with several adverse effects. ACE inhibitors can cause a dry cough, angioedema, and hyperkalemia, while ARBs may induce dizziness or hypotension. Both classes may contribute to renal dysfunction, particularly in patients with bilateral renal artery stenosis or volume depletion. The long-term immunosuppressive potential of RAAS blockers, including an increased susceptibility to infections or impaired vaccine responses, warrants further investigation and safety measures in immunocompromised or elderly individuals [2,4,77,78,79].

## 3. Clinical Implications, Emerging Applications, and Future Directions

### 3.1. Potential Benefits in Autoimmune Diseases (Multiple Sclerosis, Autoimmune Myocarditis)

RAAS modulation has demonstrated therapeutic potential in autoimmune disorders. In experimental autoimmune encephalomyelitis and a murine model of multiple sclerosis (MS), the inhibition of Ang II signaling reduced the autoreactive T-cell populations while increasing the CD4^+^ FoxP3^+^ regulatory T-cells, potentially reversing the disease progression [80,81].

Similarly, in autoimmune myocarditis, captopril attenuated myosin-specific immune responses and reduced cardiac inflammation without significantly affecting the T-cell activity or antibody production [75]. These findings suggest that ACE inhibition modulates cell-mediated immunity via mechanisms independent of direct T-cell suppression. Both captopril and losartan have demonstrated efficacy in mitigating inflammation and tissue injury in myosin-induced autoimmune models [31,82].

### 3.2. Role in Infectious Diseases

ACE plays a dual role in host immunity and inflammation. In addition to its cardiovascular functions, ACE is expressed in macrophages and neutrophils, where it facilitates MHC class I antigen presentation, enhances CD8^+^ T-cell activation, and supports the phagocytic clearance of pathogens such as *Listeria monocytogenes* and *Staphylococcus aureus* [19,39,40,44].

However, ACE also drives pro-inflammatory signaling via Ang II–AT1R activation, inducing cytokine release (TNF-α, IL-6, IFN-γ) and ROS production, potentially exacerbating inflammation in sepsis or viral infections [83]. ACEIs reduce these mediators and confer protection in experimental infection models, though they may impair antimicrobial defense, as evidenced by reduced neutrophil function and an increased bacterial load in ACE-deficient mice [2,39,73].

On the other hand, long-term immunomodulation through RAAS inhibition may impact infection susceptibility, especially in elderly or immunocompromised individuals. Prolonged ACE inhibition can impair antigen presentation and neutrophil activity, raising concerns about vaccine responsiveness and sepsis outcomes. These risks underscore the need for tailored therapy and monitoring in chronic RAAS blockades [2,73].

In COVID-19, the dysregulation of the RAAS becomes particularly relevant because SARS-CoV-2 binds to ACE2, reducing its availability and tipping the balance toward unopposed Ang II activity. This shift contributes to a cytokine storm, endothelial damage, and acute lung injuries [84,85].

Although the SARS-CoV-2-induced downregulation of ACE2 might initially appear to be protective by limiting the viral entry, the virus exhibits a significantly higher binding affinity to ACE2 compared to SARS-CoV-1, making this effect insufficient to reduce the viral spread. Instead, ACE2 depletion impairs Ang II degradation, exacerbating tissue injuries. This mechanism is particularly detrimental in individuals with a baseline ACE2 deficiency, such as the elderly or patients with hypertension, diabetes, or cardiovascular disease. Despite ACE2’s central role in COVID-19’s pathogenesis, the current evidence does not support a link between RAAS inhibitors and increased disease severity [86,87].

Several clinical studies have explored the impact of ACEIs and ARBs in COVID-19 patients [88]. An analysis of 54 randomized clinical trials, enrolling a total of 63,969 participants, found that ARBs (e.g., losartan, telmisartan) were associated with a reduced risk of severe outcomes, potentially due to their ability to block AT1Rs without affecting the ACE2 levels [89]. In a multiple-population-based case–control study in Galicia (north-west Spain), real-world data suggested that *enalapril* and *candesartan* were associated with a considerable reduction in the risk of severe COVID-19 outcomes [90]. In a retrospective study among COVID-19 patients with hypertension, the use of ACEi/ARBs was not associated with an increased risk of disease severity compared with that of patients not undergoing this treatment, suggesting that ACEi/ARBs could continue to be used as an antihypertensive therapy for COVID-19 patients, according to the recommendations of international societies [91]. The recommendations in different studies take into account the relationship between the severity of the COVID-19 infection and treatment with RAAS inhibitors and also if this treatment is ongoing or initiated at the moment of infection. Thus, a recent meta-analysis by Lee et al. (2024), involving sixteen randomized clinical trials, indicated that the use of ACEIs and ARBs may be continued in non-severe COVID-19 infections, where indicated, while the initiation of RAS blockers may be harmful in critically ill patients [92]. Other studies, however, report slightly different findings, showing that no significant correlation was identified between ACEi/ARB use and the risk of severe COVID-19 outcomes, including intensive care admission, the need for mechanical ventilation, or mortality [93,94,95].

### 3.3. Impact on Cancer Immunology and Tumor Microenvironment

The RAAS significantly influences tumor biology. Ang II–AT1R signaling promotes proliferation, angiogenesis, and resistance to apoptosis, whereas the Ang(1–7)-Mas and Ang II–AT2R pathways exert counter-regulatory, antitumor effects. RAAS components are expressed by tumor and stromal cells, driving angiogenesis and supporting inflammation via immune-derived cytokines and ROS [43]. The Ang(1–7)-Mas axis acts via the Mas receptor, a G protein-coupled receptor suppressing AT1R signaling by enhancing eNOS activation, suppressing NF-κB, and promoting anti-inflammatory cytokines like IL-10 [96].

Ang II stimulates vascular endothelial growth factor A (VEGF-A) expression through the epidermal growth factor receptor (EGFR), mitogen-activated protein kinase (MAPK), extracellular signal-regulated kinase (ERK1/2), and PI3K/AKT pathways, facilitating neovascularization in various cancers [97]. Additional oncogenic cascades activated by Ang II include PAX2, STAT3, and JAK2 in prostate cancer [98] and RAS/RAF/ERK1/2 in gastric malignancies [99].

The effects of RAAS blockers on the cancer risk and progression appear to be cancer-type-specific. Clinical trials have linked the use of RAAS blockers to a decreased risk of colorectal, keratinocyte, and prostate cancers, while the associations with liver and breast cancers remain inconclusive [100,101,102]. Other studies reported increased risks of bladder and kidney cancers [100], and the long-term use of ACEIs has been associated with a higher risk of lung cancer, potentially due to bradykinin and substance P accumulation [103].

However, when it comes to cancer survival, RAAS blockers appear to exert beneficial effects, especially when combined with chemotherapy. Significant survival advantages have been observed in breast, colorectal, pancreatic, hepatocellular, and prostate cancers and non-small-cell lung cancer (NSCLC) [104,105,106,107]. A large cohort study involving 73,170 breast cancer patients found that ARBs’ use was associated with reduced breast cancer–specific mortality, both pre- and post-diagnosis, in a dose-dependent manner [105].

Furthermore, ACEIs such as captopril have demonstrated anti-tumor effects in colorectal cancer, reducing the tumor viability and metastasis while altering the T-cell profiles in the tumor microenvironment. Captopril increased the CD4^−^CD8^−^ double-negative T-cells and reduced the CD4^+^ T-cells without affecting CD8^+^ populations, suggesting a complex immunomodulatory role [1,108].

These findings underscore the potential of RAAS blockers for use as adjuncts in cancer therapy, where they may suppress tumor growth, enhance apoptosis, and improve drug delivery [106,107,109]. Notably, RAAS blockers also mitigate chemotherapy-induced cardiotoxicity, particularly in breast cancer patients treated with trastuzumab or anthracyclines (Table 4), as demonstrated in multiple clinical studies [110,111,112].

### 3.4. Potential Benefits in Neurodegenerative Diseases

Emerging evidence highlights a potential role for ACE in treating Alzheimer’s disease (AD) due to its capacity to degrade amyloid beta 1–42 (Aβ1–42). In transgenic AD mice overexpressing ACE in myelomonocytic cells, enhanced Aβ clearance, a reduced plaque burden, attenuated astrogliosis, and preserved cognitive function were demonstrated, driven by macrophage-mediated phagocytosis. These findings suggest that ACE contributes to neuroprotection through immunomodulation and Aβ metabolism [37]. While ACE inhibition may counteract these effects, some clinical studies have reported short-term cognitive benefits from ACEIs in early Alzheimer’s cohorts, underscoring the need for the context-specific evaluation of RAAS modulation in neurodegenerative disease management [113,114,115].

Centrally acting ACEIs (C-ACEIs), such as perindopril, fosinopril, trandolapril, and ramipril, have garnered increasing attention in AD research due to their potential neuroprotective properties and ability to cross the blood–brain barrier [10,11]. Observational studies have suggested that these agents may slow cognitive decline compared to non-centrally acting ACEIs (NC-ACEIs) (enalapril, lisinopril, benazepril) and calcium channel blockers [114,115]. While evidence on the dementia incidence remains inconclusive, recent clinical studies support the use of C-ACEIs to mitigate cognitive deterioration in older adults with hypertension [116,117]. Also, among hypertensive patients with mild cognitive impairment, ARBs were associated with a lower dementia risk compared to ACEIs and other antihypertensives, although these findings require validation in larger prospective studies [118].

A large-scale study examined the cognitive trajectories and survival in patients receiving C-ACEIs, NC-ACEIs, or no ACEIs. The results showed a modest improvement in cognitive performance within the first nine months among the C-ACEI users, whereas those on NC-ACEIs experienced a decline. However, no long-term differences in the cognitive outcomes or survival were observed, highlighting a potential short-term benefit that warrants further longitudinal evaluation [113].

The mechanisms underlying these C-ACEI-mediated cognitive effects have not been fully elucidated. Although some preclinical studies suggest that ACE inhibition may impair amyloid beta (Aβ1–42) clearance [37], other findings support a neuroprotective role through enhanced cerebral perfusion, reduced neuroinflammation, and improved cholinergic function [117,119,120]. C-ACEIs may counteract Ang II–induced vasoconstriction, downregulate pro-inflammatory cytokines, and increase the acetylcholine availability, possibly synergizing with acetylcholinesterase inhibitors [113]. Perindopril has been shown to provide hippocampal ACE inhibition and cognitive protection in murine AD models [121]. Taking these results together, C-ACEIs may offer a clinical benefit in selected AD patients, particularly in the early stages or when combined with standard cognitive therapies (Table 5) [122,123].

## 4. Conclusions

RAAS inhibitors, traditionally used for the treatment of cardiovascular and renal indications, exhibit significant immunomodulatory properties with therapeutic relevance across a wide spectrum of diseases. Through both Ang II-dependent and -independent pathways, ACEIs and ARBs influence innate and adaptive immune mechanisms, including cytokine regulation, antigen presentation, and T-cell polarization. Evidence supports their potential to attenuate chronic inflammation, modulate autoimmune responses, and enhance antitumor immunity. Centrally acting ACEIs may also confer cognitive protection in neurodegenerative disorders, while ARBs demonstrate unique anti-inflammatory effects via PPARγ activation. Despite the promising preclinical and clinical findings, the immunological impact of RAAS modulation is complex and context-dependent. Future research should aim to determine the long-term effects of ACEIs and ARBs across diverse pathological settings and explore their integration into immunomodulatory treatment strategies beyond their conventional cardiovascular use.

## Figures and Tables

**Table 1:** Pharmacological classification of RAAS inhibitors.

Class	Examples	Mechanism of Action
ACE inhibitors (centrally acting—C-ACEIs)	Perindopril, ramipril, trandolapril	Cross the blood–brain barrier (lipophilic); may affect the central RAAS and neuroinflammation [10,15]
ACE inhibitors (non-centrally acting—NC-ACEIs)	Enalapril, lisinopril, benazepril	Do not significantly penetrate the CNS; act primarily peripherally [11,12]
Angiotensin II receptor blockers (ARBs)	Losartan, telmisartan, valsartan, candesartan	Block AT1 receptors; some (e.g., telmisartan) also activate PPARγ [13,16]
Direct renin inhibitors	Aliskiren	Inhibit conversion of angiotensinogen to Ang I; rarely used alone [17]
Mineralocorticoid receptor antagonists (MRAs)	Spironolactone, eplerenone	Block aldosterone receptors; reduce sodium retention and fibrosis [14]

CNS—central nervous system.

**Table 4:** Comparative effects of ACEIs and ARBs regarding cancer biology, immunology, and progression.

Domain	ACEIs	ARBs
Tumor cell biology	Reduce proliferation and metastasis; induce apoptosis (e.g., captopril in colorectal cancer) [108]	Suppress tumor growth and angiogenesis [109]
Angiogenesis	Indirect inhibition via decreased Ang II levels [108]	Direct inhibition via AT1R blockade; suppress VEGF-A expression [98]
Immune modulation	Modify tumor microenvironment: ↑CD4^−^CD8^−^, ↓CD4^+^ T-cells; complex immunoregulatory effects [108]	Less clearly defined; potential anti-inflammatory role
Cancer risk	Increased risk of lung, bladder, and kidney cancers (long-term use) [100,103]	Decreased risk of colorectal, keratinocyte, and prostate cancers [101,102]
Cancer survival	Improve survival, particularly when combined with chemotherapy [104,107]	Associated with reduced cancer-specific mortality; dose–response effect seen in breast cancer [105]
Cardiotoxicity protection	Mitigate cardiotoxicity from anthracyclines and trastuzumab [110,111,112]	Similar cardioprotective effects in chemotherapy settings [110,111,112]

**Table 5:** Comparative effects of ACEIs and ARBs in neurodegenerative disorders.

Domain	ACEIs	ARBs
Amyloid beta clearance	Inhibit ACE-mediated Aβ1–42 degradation; potential reduction in clearance [37]	Not directly involved in Aβ degradation
Cognitive outcomes	C-ACEIs (e.g., perindopril, ramipril) shown to provide short-term improvement; NC-ACEIs less effective [113]	Lower dementia risk versus that with ACEIs in hypertensive patients with mild cognitive impairment [118]
Neuroinflammation	Reduce pro-inflammatory cytokines; potential neuroprotective effect [120]	May exert anti-inflammatory effects via AT1R blockade [120]
Cerebral perfusion	Improve cerebral blood flow by inhibiting Ang II-mediated vasoconstriction [121]	Reduce vasoconstriction via AT1R blockade [118,120]
Cholinergic function	Enhance acetylcholine release; may synergize with cholinesterase inhibitors [113]	Effect on cholinergic transmission not well established
Clinical implications	Potential early-stage benefit in AD; recommended in hypertensive older adults [113]	Lower dementia risk in hypertensive patients with mild cognitive impairment [118]
